# Changes in HIV Incidence among People Who Inject Drugs in Taiwan following Introduction of a Harm Reduction Program: A Study of Two Cohorts

**DOI:** 10.1371/journal.pmed.1001625

**Published:** 2014-04-08

**Authors:** Yen-Fang Huang, Jyh-Yuan Yang, Kenrad E. Nelson, Hsu-Sung Kuo, Chin-Yin Lew-Ting, Chin-Hui Yang, Chang-Hsun Chen, Feng-Yee Chang, Hui-Rong Liu

**Affiliations:** 1 Centers for Disease Control, Ministry of Health and Welfare, Taipei, Taiwan; 2 Institute of Health Policy and Management, College of Public Health, National Taiwan University, Taipei, Taiwan; 3 Department of Epidemiology, Bloomberg School of Public Health, Johns Hopkins University, Baltimore, Maryland, United States of America; 4 Department of Public Health, College of Public Health, National Taiwan University, Taipei, Taiwan; San Francisco Department of Public Health, United States of America

## Abstract

**Background:**

Harm reduction strategies for combating HIV epidemics among people who inject drugs (PWID) have been implemented in several countries. However, large-scale studies using sensitive measurements of HIV incidence and intervention exposures in defined cohorts are rare. The aim of this study was to determine the association between harm reduction programs and HIV incidence among PWID.

**Methods and Findings:**

The study included two populations. For 3,851 PWID who entered prison between 2004 and 2010 and tested HIV positive upon incarceration, we tested their sera using a BED HIV-1 capture enzyme immunoassay to estimate HIV incidence. Also, we enrolled in a prospective study a cohort of 4,357 individuals who were released from prison via an amnesty on July 16, 2007. We followed them with interviews at intervals of 6–12 mo and by linking several databases. A total of 2,473 participants who were HIV negative in January 2006 had interviews between then and 2010 to evaluate the association between use of harm reduction programs and HIV incidence. We used survival methods with attendance at methadone clinics as a time-varying covariate to measure the association with HIV incidence. We used a Poisson regression model and calculated the HIV incidence rate to evaluate the association between needle/syringe program use and HIV incidence. Among the population of PWID who were imprisoned, the implementation of comprehensive harm reduction programs and a lower mean community HIV viral load were associated with a reduced HIV incidence among PWID. The HIV incidence in this population of PWID decreased from 18.2% in 2005 to 0.3% in 2010. In an individual-level analysis of the amnesty cohort, attendance at methadone clinics was associated with a significantly lower HIV incidence (adjusted hazard ratio: 0.20, 95% CI: 0.06–0.67), and frequent users of needle/syringe program services had lower HIV incidence (0% in high NSP users, 0.5% in non NSP users). In addition, no HIV seroconversions were detected among prison inmates.

**Conclusions:**

Although our data are affected by participation bias, they strongly suggest that comprehensive harm- reduction services and free treatment were associated with reversal of a rapidly emerging epidemic of HIV among PWID.

*Please see later in the article for the Editors' Summary*

## Abstract 2

Kenrad Nelson and colleagues report on the association between HIV incidence and exposure to a national harm-reduction program among people who inject drugs in Taiwan.

*Please see later in the article for the Editors' Summary*

## Editors' Summary

**Background:**

About 35 million people worldwide are currently infected with HIV, the virus that causes AIDS, and about 2.3 million people become newly infected every year. HIV is mainly transmitted through unprotected sex with an infected partner. However, people who inject drugs (PWID) have a particularly high risk of HIV infection because blood transfer through needle and syringe sharing can transmit the virus. It is estimated that 5%–10% of all people living with HIV are PWID. Indeed, in some regions of the world the primary route of HIV transmission is through shared drug injection equipment and the prevalence (the proportion of a population that has a specific disease) of HIV infection among PWID is very high. In Asia, for example, more than a quarter of PWID are HIV positive. Because the high prevalence of HIV among PWID poses a global health challenge, bodies such as the Joint United Nations Programme on HIV/AIDS endorse harm reduction strategies to prevent risky injection behaviors among PWID. These strategies include the provision of clean needles and syringes, opioid substitution therapy such as methadone maintenance treatment, and antiretroviral treatment for HIV-positive PWID.

**Why Was This Study Done?:**

Although harm reduction strategies for combating HIV epidemics among PWID have been implemented in several countries, few large-scale studies have examined the association between HIV incidence (the proportion of new cases of HIV in a population per year) and exposure to harm reduction programs among PWID. In this cohort study (an investigation that determines the characteristics of a group of people and then follows them over time), the researchers determine the association between harm reduction programs and HIV incidence among PWID in Taiwan. HIV infections used to be rare among the 60,000 PWID living in Taiwan, but after the introduction of a new HIV strain into the country in 2003, an HIV epidemic spread rapidly. In response, the Taiwanese government introduced a pilot program of harm reduction that included the provision of clean needles and syringes and health education in July 2005. The program was expanded to include methadone maintenance treatment in early 2006 and implemented nationwide in June 2006.

**What Did the Researchers Do and Find?:**

The researchers enrolled two study populations. The first cohort comprised 3,851 PWID who were incarcerated for illicit drug use between 2004 and 2010 and who tested positive for HIV upon admission into prison. By using the BED assay, which indicates whether an HIV infection is recent, the researchers were able to determine the HIV incidence among the prisoners. In 2004, the estimated HIV incidence among prisoners with a history of drug injection was 6.44%. The incidence peaked in 2005 at 18.2%, but fell to 0.3% in 2010.

The second study population comprised 2,473 individuals who were HIV negative on January 1, 2006, and who had been incarcerated for drug use crimes but were released on July 16, 2007, during an amnesty. The researchers regularly interviewed these participants between their release and 2010 about their use of harm reduction interventions, and obtained other data about them (for example, diagnosis of HIV infection) from official databases. Analysis of all these data indicated that, in this cohort, attendance at methadone maintenance treatment clinics and frequent use of needle and syringe services were both associated with a significantly lower HIV incidence.

**What Do These Findings Mean?:**

These findings suggest that the introduction of a comprehensive harm reduction program in Taiwan was associated with a significant reduction in the HIV incidence rate among PWID. These findings must be interpreted with caution, however. First, because the participants in the study were selected from PWID with histories of incarceration, the findings may not be representative of all PWID in Taiwan or of PWID in other countries. Second, PWID who chose to use needle and syringe services or methadone maintenance treatment clinics might have shared other unknown characteristics that affected their risk of HIV infection. Finally, some of the reduction in HIV incidence seen during the study is likely to be associated with the availability of free treatment, which has been offered to all HIV-positive individuals in Taiwan since 1997. Despite these limitations, these findings suggest that countries with a high prevalence and incidence of HIV among PWID should provide comprehensive harm reduction services to their populations to reduce risky drug injection behaviors.

**Additional Information:**

Please access these websites via the online version of this summary at http://dx.doi.org/10.1371/journal.pmed.1001625.

- Information is available from the US National Institute of Allergy and Infectious diseases on HIV infection and AIDS
- NAM/aidsmap provides basic information about HIV/AIDS, and summaries of recent research findings on HIV care and treatment
- Information is available from Avert, an international AIDS charity, on many aspects of HIV/AIDS, including information on injecting drug users and HIV/AIDS and on harm reduction and HIV prevention (in English and Spanish)
- The US National Institute on Drug Abuse also provides information about drug abuse and HIV/AIDS (in English and Spanish)
- The 2013 UNAIDS World AIDS Day report provides up-to-date information about the AIDS epidemic and efforts to halt it
- Personal stories about living with HIV/AIDS are available through Avert, Nam/aidsmap, and Healthtalkonline

## Introduction

The high prevalence of HIV among people who inject drugs (PWID) in many countries represents a global health challenge [1]. In response to this problem, harm reduction strategies have been endorsed by the Joint United Nations Programme on HIV/AIDS (UNAIDS), the United Nations Office on Drugs and Crime, and the World Health Organization [2]. Core prevention interventions for risky injection behavior include the following: needle and syringe programs (NSPs), i.e., the provision of clean needles and syringes; opioid substitution therapy (OST), such as methadone maintenance treatment (MMT); and antiretroviral therapy (ART) for HIV-positive PWID [2],[3]. Several studies have demonstrated an association between these interventions and decreased HIV incidence, but review papers have indicated that many of these studies were limited by the use of only self-reports of risky behavior, had short follow-up periods, or reported high attrition rates. Large-scale studies using good measurements of HIV incidence and intervention exposures in defined cohorts have been rare [4]–[7].

The other important issue regarding the implementation of these harm reduction programs to reduce the transmission of HIV among PWID is whether or not the services are readily available and user friendly to the target population [5],[8]. Country-level comparisons and modeling projections in Western countries have shown that the extent of coverage of OST and the provision of clean needles and syringes are associated with the incidence and prevalence of HIV among PWID [4],[9]–[11]. Several reports of successful implementation of comprehensive harm reduction programs to control the transmission of HIV among PWID have been published [1]–[6],[9]–[11]. However, these studies have rarely measured the association of the components of comprehensive harm reduction services and HIV incidence at an individual level. In Taiwan a comprehensive harm reduction program was designed and implemented during an extensive HIV epidemic that had already spread among PWID in this country, providing an opportunity to measure the association between harm reduction strategies and HIV incidence.

There are an estimated 60,000 PWID in Taiwan, among whom diagnosed HIV infections remained low until 2003, when an epidemic of HIV began to spread rapidly after an HIV recombinant virus, CRF 07_BC, was introduced into the PWID population [12],[13]. In 2004, the Taiwanese government sought advice from experts abroad to develop harm reduction programs for PWID to control the epidemic. A pilot program, including NSP and health education, was started in four of Taiwan's 23 administrative areas in July 2005, and MMT was introduced in February 2006. The programs were implemented nationwide in June 2006 [13],[14]. In 2012, there were 102 MMT clinics, 929 NSP stations, and 415 needle/syringe vending machines in Taiwan. The Taiwanese government has offered free access to ART to HIV-positive individuals since 1997, including HIV-infected persons in prison [15]. One recent report showed that these extensive harm reduction programs, especially health education, have been associated with a reduction in the number of new HIV cases reported among PWID in Taiwan; however, this ecological study had important limitations [16]. An ecological study cannot detect individual-level biological effects, and the number of newly reported cases was not equal to the true HIV incidence because of incomplete detection and reporting. Data from the BED HIV-1 capture enzyme immunoassay (BED assay) can provide some insight into the true recent incidence of HIV infection [17],[18]. In this study, we tested all newly detected HIV infections in prisoners who were PWID with BED assays, and we also initiated a systematic follow-up of a cohort of former prisoners who had been imprisoned for crimes related to illegal drug use and who were released from prison on July 16, 2007. The aims of this study were to explore the following: (1) the temporal trend in HIV incidence among PWID before and after the harm reduction program, (2) the associations between harm reduction strategies and HIV seroconversion, and (3) the protective and risk factors for HIV infection among PWID.

## Methods

### Ethics Statement

The research included two studies. In the population-level study, the serum samples were compulsory (required to be collected by law). In the individual-level study, participants provided written informed consent. Both studies were approved by the Institutional Review Board Committee of the Taiwan Centers for Disease Control (CDC).

### Settings

The first study involved analyzing BED assay results to detect recent HIV infections among 3,851 prisoners who were incarcerated for illicit drug use in Taiwan and were newly diagnosed with HIV. The Drug Prevention and Control Act in Taiwan requires drug offenders with a positive urine drug test to undergo 3–6 mo of a rehabilitative detoxification intervention in a correctional facility. Prison terms are usually 1 to 5 y for a second offense of schedule 1 drug use. Schedule 1 substances include heroin, morphine, opiates, and cocaine. Heroin was the drug used in more than 95% of schedule 1 drug use arrests in Taiwan. In Taiwan, a nationwide active surveillance system for HIV/AIDS was established by law in 1989, including mandatory HIV testing for new inmates upon admission into prison and on a yearly basis among prisoners from 1990 onwards, to monitor the HIV epidemic in the drug-using population.

The second study followed a cohort of 4,357 participants who had been incarcerated for drug use crimes and were amnestied from prison on July 16, 2007 [14]. It is possible to effectively trace citizens of Taiwan since all persons are issued a personal identification number at birth, and all subsequent life events, including incarceration and medical data, are linked to this number. In addition, all HIV test results are required to be reported to the CDC, and aliquots of sera from these patients are provided to the CDC by law. Emigration of persons with a history of drug use is extremely rare, so follow-up of these participants is quite feasible.

### Participants and Ethics

According to the official report from the Ministry of Justice in Taiwan, the number of prisoners who were incarcerated annually for drug use offenses between January 1, 2004, and December 31, 2010, ranged from 15,630 to 34,017 (Table 1) [19]. The number of HIV cases reported annually among drug users in prison ranged from 511 in 2004 to a peak of 2,040 in 2005, with a low of 82 in 2010 [20]. A total of 3,851 (74%) surplus serum samples from 5,222 HIV-positive drug users were collected to estimate the HIV incidence among PWID in prison from 2004 to 2010. The serum samples were required to be collected by law. However, since PWID often have damaged veins, the volume of blood collected in 2004 and the early period of 2005 was sometimes inadequate to do BED assays on the available serum. However, this problem was corrected after 2005. There were no differences in the age and gender distributions of people diagnosed with HIV with and without a BED assay.

**Table 1:** The distribution of prisoners with histories of drug use and their HIV status.

Year	Number of Prisoners with a History of Drug Use	New HIV Diagnoses in PWID	Reported HIV Cases among Inmates with a History of Drug Use		Cases with Completed BED Assay		Cases of Recent Infection		HIV Incidence^a^	
			Number	Percent	Number	Percent	Number	Percent	Estimate	95% CI
2004	15,630	620	511	82%	115	23%	103	90%	6.44%	5.09%–7.78%
2005	19,293	2,420	2,040	84%	1,363	67%	926	68%	18.16%	16.38%–19.96%
2006	20,396	1,839	1,622	88%	1,413	87%	878	62%	11.58%	10.42%–12.74%
2007	27,715	742	573	77%	545	95%	248	46%	1.84%	1.55%–2.13%
2008	34,017	386	269	70%	244	91%	113	46%	0.85%	0.68%–1.01%
2009	25,437	178	125	70%	107	86%	36	34%	0.29%	0.17%–0.41%
2010	21,338	116	82	71%	64	78%	25	39%	0.27%	0.15%–0.40%

a The estimated HIV incidence among PWID was from tested sera from 3,851 prisoners who were incarcerated because of illicit drug use who were diagnosed with HIV between 2004 and 2010; the sera were tested using a modified BED assay to estimate HIV incidence according to UNAIDS and World Health Organization guidelines.

**Figure 1:**
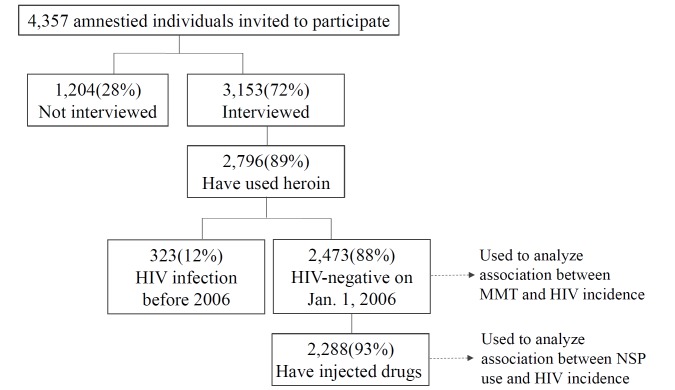
Flow diagram of all participants in the amnesty cohort and their drug-related behavior.

The 3,153 participants in the amnesty cohort were contacted, interviewed, and tested for HIV antibodies after their amnesty; 2,796 participants (89%) reported ever using heroin. Participants were interviewed four times at intervals of 6–12 mo between January 1, 2007, and December 31, 2010. In addition, their individual registration and attendance at methadone clinics throughout the island was recorded and reported to the CDC. For individual-level analysis of the association of harm reduction strategies with HIV seroconversion, we identified from among the 3,153 participants with recorded interviews 2,473 participants who reported ever using heroin and who were HIV negative on January 1, 2006; of these potential study participants, 2,288 (93%) had injected heroin. We were able to link the HIV reporting system, the correctional database, and the interview dataset to identify these study participants (Figure 1). The follow-up period for each participant in the study was from the beginning of 2006 to the end of 2010. However, the participants had different follow-up periods between 2008 and 2010 for some of the questions about use of NSPs because some of these questions were changed during the study to improve our understanding of the possible association of NSP use and HIV incidence. All personal identifying information was removed after the record linkage was completed.

### Measurements

In the national drug offender population, the HIV status of each individual was assessed by BED assay to distinguish between recent and long-term infections. The BED assay is an assay developed by scientists at the US Centers for Disease Control and Prevention and utilized to distinguish more recent from chronic seroprevalent HIV infections [21]. The assay contains HIV antigens from HIV serogroups B, E, and D, hence the name. The BED assay detects the level of anti-HIV IgG relative to total IgG and is based on the fact that the ratio of anti-HIV IgG to total IgG increases with time after HIV infection. Initial testing for all specimens is done for a single sample; specimens with an ODn (optical density) of <1.2 are tested in triplicate to confirm ODn status. If the ODn of the specimen is <0.8, the specimen is considered a recent seroconversion. Harm reduction services were recorded as the number of syringes dispatched nationally to NSP sites and the number of person-days of attendance at MMT. The population plasma HIV-1 RNA concentration among PWID with HIV was also recorded in the national HIV/AIDS management system.

In the amnesty cohort, since harm reduction services were not available in prison, person-days for which participants were in prison were not included in the analysis. The number of person-days of attendance at MMT was obtained from the daily MMT records in the national MMT system database. MMT dropouts were defined as participants who were absent and who stopped taking methadone for 14 or more consecutive days. Individuals could re-enroll in MMT if they dropped out during this study. All of the MMT clinics and hospitals provided information on all of the MMT patients, including their daily attendance records.

For estimating NSP use, attendance at any NSP site was recorded via interview. The three major types of NSPs in Taiwan are pharmacy-based NSPs (70%), local health stations (10%), and vending machines (10%). Pharmacy-based NSPs and local health stations exchange clean needles/syringes for used needles/syringes turned in by PWID. The exchange ratio for clean/used needles/syringes is 1∶1, and there are no limits on the numbers of needles and syringes that can be exchanged. We asked the participants at each follow-up interview what proportion of injection events they used clean syringes and needles for injecting drugs in the past 6 mo, and, additionally in the second, third, and fourth interviews, where they obtained clean injection equipment. Syringe vending machines were available throughout Taiwan starting in 2008. We added a question about vending machine use to interviews conducted in 2009 and 2010. We defined NSP use groups by vending machine use (yes/no) and NSP use (high/low). We further defined the following subgroups for NSP use between 2008 and 2010: non NSP users (never using NSP services), high NSP users (more than 60% of needles/syringes from NSPs per 100 injecting events, and never buying needles/syringes from vending machines), vending machine users (ever buying needles/syringes from vending machines), and low NSP users (less than 60% of needles/syringes from NSPs per 100 injecting events, and never buying needles/syringes from vending machines). No PWID who reported ever buying needles/syringes from vending machines reported more than 60% of NSP coverage when they injected drugs.

### Statistical Analysis

The annual HIV incidence among PWID prisoners was calculated using the following formula, according to UNAIDS and World Health Organization guidelines, to estimate the HIV incidence at a population level [18],[22]:
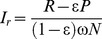
(1)where *I_r_* is HIV incidence, *N* is the number of HIV-negative individuals in the survey, *P* is the number of HIV-positive individuals in the survey, and *R* is the number of people classified as positive by the recent infection testing algorithm (RITA), excluding individuals diagnosed with AIDS concurrently or having HIV infection for more than 6 mo after HIV diagnosis. ω is the mean RITA duration in years (assumed to be 176 d in this study [23]). ε is the false recent rate of the RITA. The false recent rate by BED assay was 7.46% among 791 samples from PWID who had a documented positive HIV test more than 12 mo previous to this study. Additionally, we also followed the UNAIDS guidelines in using the following formula to handle HIV-positive samples with missing RITA tests:
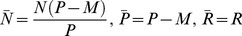
(2)where *M* is the number of HIV-positive samples with a missing RITA test, and *N*, *P*, and *R* defined as above.

To calculate HIV incidence in the cohort, follow-up continued to the date of HIV diagnosis or death; the data were censored on December 31, 2010, for participants who were not HIV-positive at that time.

To evaluate the association of MMT and HIV incidence, we constructed a Cox regression model with MMT attendance as a time-varying covariate, using delayed-entry techniques (left truncation) to account for potential immortal-time bias [24]. We also adjusted for age, sex, education, and number of incarcerations before 2006. To evaluate the association of NSP use and HIV incidence, we constructed a Poisson regression model with NSP use as a time-varying covariate and adjusted for the MMT variable. In addition, we calculated the HIV incidence for NSP use subgroups. Because the HIV incidence rate tended to be near 0 in some NSP subgroups, the 95% confidence interval for HIV incidence for each subgroup was calculated as a Wilson score confidence interval [25]. Analyses were conducted using SAS, version 9.2, and Microsoft Excel 2010.

## Results

### Description of the Amnesty Cohort

Among the 2,473 individuals in the prison amnesty cohort who reported ever using heroin in interviews and who were HIV-negative in 2006, 90% were male. In the year of entry into the study (2006), the median age was 29 y (interquartile range: 24–34), and the median number of years of education was 9 y (interquartile range: 9–11). Most participants had a previous prison history before the incarceration for which they were amnestied in July 2006, and the median number of incarcerations before 2006 was three (interquartile range: 2–4) (Table 2). The average time from the onset of injecting drug use to first imprisonment was 5.4 y.

**Table 2:** Characteristics of injecting drug users enrolled in the cohort in 2006.

Characteristic	Number (*n* = 2,473) or Median (25%–75%)	Percent
**Age in 2006**		
<20 y	248	10%
20–29 y	1,158	47%
30–39 y	826	33%
≧40 y	241	10%
Median (25%–75%)	29 (24–34)	
**Sex**		
Male	2,232	90%
Female	241	10%
**Education (median [25%–75%])**	9 (9–11)	
**Number of incarcerations before 2006**		
0	33	1%
1	300	12%
2	852	34%
3	554	22%
4	422	17%
5	214	9%
6	69	3%
≧7	29	1%
Median (25%–75%)	3 (2–4)	
**Ever MMT**		
No	1,001	40%
Yes	1,472	60%
**Ever NSP use**		
No	1,627	66%
Yes	846	34%
**Utilization of harm reduction**		
Never	774	31%
Only NSP	227	9%
Only MMT	853	35%
NSP and MMT	619	25%
**Number of HIV seroconversions during each year of follow-up**	51	2%
2006	16	
2007	11	
2008	18	
2009	4	
2010	2	

By December 31, 2010, a cumulative total of 1,699 (69%) individuals had ever attended harm reduction sites, among whom 1,472 (60%) had been to MMT sites, and 846 (34%) had been to NSP sites. Among the 2,473 individuals followed for 12,140.1 person-years, 51 seroconverted (Table 2). 2,400 (95%) participants were incarcerated twice or more during the follow-up period: at each readmission to prison they were tested again for HIV antibody. The average time in prison for the 2,473 participants between 2006 and 2010 was 836 d.

### HIV Epidemic among PWID and the Coverage of Harm Reduction in the Prisoner Population Study

**Figure 2:**
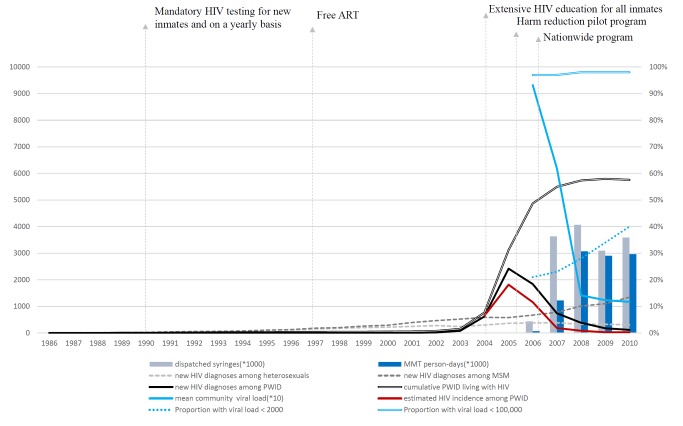
The temporal trend in HIV incidence among PWID and distribution of harm reduction services. Annual number of dispatched syringes is the total number of clean needles/syringes purchased by the CDC. The number of MMT person-days was calculated from personal records from the national methadone management system. Data on the number of new HIV diagnoses via different transmission routes, the cumulative number of PWID living with HIV, and community viral load were from the national HIV/AIDS reporting system. The estimated HIV incidence among PWID was from tested sera from 3,851 prisoners who were incarcerated because of illicit drug use who were diagnosed with HIV between 2004 and 2010; the sera were tested using a modified BED assay to estimate HIV incidence. Viral load values are copies/milliliter. MSM, men who have sex with men.

A total of 3,851 sera were available from HIV-positive drug users in the national prisoner population, among which 2,329 (60%) were recent infections. The number and proportion of all cases that the BED assay indicated were recent infections, by year, was 103 (90%) in 2004, 926 (68%) in 2005, 878 (62%) in 2006, 248 (46%) in 2007, 113 (46%) in 2008, 36 (34%) in 2009, and 25 (39%) in 2010. In 2004, the estimated HIV incidence among prisoners with histories of drug use (heroin mostly) in Taiwan was 6.44% (95% CI: 5.09%–7.78%). The incidence peaked (18.16%, 95% CI: 16.38%–19.96%) in 2005 and decreased starting in 2006. In 2007, HIV incidence (1.85%, 95% CI: 1.55%–2.13%) was significantly lower than in 2004 (Table 1), when more than 3,600,000 syringes (an average of 60 syringes per PWID per year) were distributed, and 5,585 persons (1,225,000 person-days) were receiving methadone. In 2008, 3,600,000 syringes were distributed, and MMT coverage peaked at more than 3,000,000 person-days (12,598 persons) and then plateaued. HIV incidence decreased to 0.27% (95% CI: 0.15%–0.40%) in 2010. The estimated temporal trend in HIV incidence among PWID was similar to the trend in annually reported HIV cases among PWID (Figure 2).

During the period of this study, the annual mean community plasma HIV-1 RNA concentration (viral load) among PWID with HIV infection was 93,153 copies/ml in 2006, 61,853 copies/ml in 2007, 14,145 copies/ml in 2008, 12,357 copies/ml in 2009, and 11,710 copies/ml in 2010. The proportion of HIV-infected PWID with an HIV viral load <2,000 copies/ml increased from 21% in 2006 to 40% in 2010. PWID with very high HIV viral load, i.e., >100,000 copies/ml, were very unusual at any time (Table 3). The rank correlation for the mean community viral load and the annual estimated HIV incidence was high (*R* ^2^ = 0.78).

**Table 3:** Community viral load among HIV-infected PWID in Taiwan.

Year	Number of PWID Who Were HIV Positive	Number on Medical Service^a^	Number on ART	Number with Records of CD4 and Viral Load Data		Proportion with CD4<350 Cells/µl	Mean Viral Load (Copies/ml)	Proportion with Viral Load <2,000 Copies/ml	Proportion with Viral Load <100,000 Copies/ml
				Number	Percent				
2006	4,933	3,558	430	2,947	60%	36%	93,153	21%	97%
2007	5,564	4,182	525	3,878	70%	22%	61,853	23%	97%
2008	5,824	4,341	582	3,955	68%	22%	14,145	28%	98%
2009	5,764	4,451	713	4,220	73%	24%	12,357	34%	98%
2010	5,738	4,339	1,067	4,019	70%	26%	11,710	40%	98%

a PWID who were HIV positive who attended a designated AIDS/HIV clinic.

HIV prevalence among PWID increased substantially from 2004 to 2005, and the rate of increase remained steep from 2005 to 2006. The rate of increase in HIV prevalence slowed between 2007 and 2009. In 2010, the total number of prevalent cases living with HIV among PWID in the community was slightly less than in 2009.

### HIV Seroconversions Associated with Incarceration

No seroconversions were detected among PWID who were in prison during the follow-up period. The self-reported frequency of injecting drugs in the cohort while they were in prison was very low. Only 3 (0.2%) of 1,704 individuals who answered the question reported that they had injected drugs when they were in prison between 2006 and 2010. The Cox proportional hazard model indicated that more frequent incarceration before 2006 (hazard ratio: 1.199, 95% CI: 0.991–1.451) was not a significant risk factor for HIV infection after controlling for other variables including sex, education, and MMT use (Table 4).

**Table 4:** Factors associated with HIV infection from a time-varying Cox regression model.

Factor	Crude Hazard Ratio	Adjusted Hazard Ratio	*p*-Value
**MMT status** a			0.0090
Not attending MMT	1	1	
Attending MMT	0.20 (0.06–0.65)	0.20 (0.06–0.67)	
**Sex**			0.6614
Male	1	1	
Female	1.06 (0.45–2.49)	1.22 (0.51–2.93)	
**Education (years)**	0.71 (0.39–1.28)	0.96 (0.85–1.09)	0.5483
**Age in 2006 (years)**	1.65 (0.95–2.86)	1.03 (0.99–1.06)	0.1487
**Number of incarcerations before 2006**	1.99 (1.14–3.48)	1.20 (0.99–1.45)	0.0615

95% confidence intervals given in parentheses.

a MMT treated as a time-varying covariate.

### HIV Seroconversions Associated with MMT Utilization

**Figure 3:**
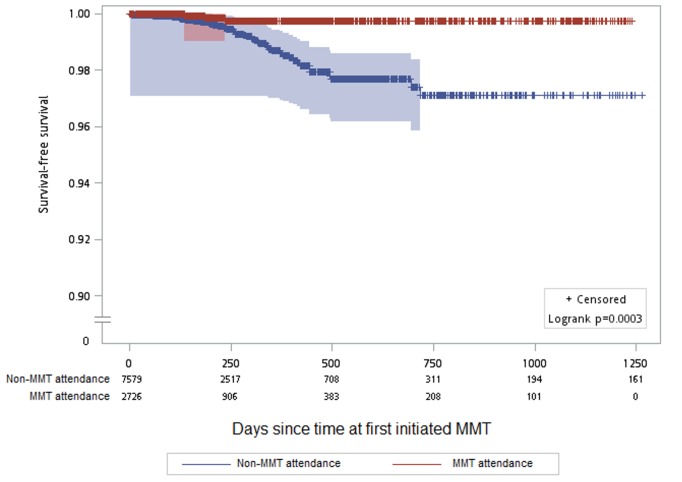
Kaplan-Meier survival estimates for HIV incidence among participants attending versus not attending MMT clinics. There were 2,473 participants followed for 12,140.1 person-years at risk on the *x*-axis. Gray shading indicates the 95% confidence interval. *y*-Axis has been interrupted to show the results in greater detail.

In the community, only three seroconversions occurred while the participants were attending MMT (HIV incidence rate = 1.65/1,000 person-years), and 48 occurred among participants while not attending an MMT site (HIV incidence rate = 10.33/1,000 person-years) (Table 5). The Kaplan-Meier survival analysis for HIV incidence among individuals attending and not attending MMT is shown in Figure 3. The Cox proportional hazard model indicated that MMT attendance was associated with a significantly lower HIV incidence (hazard ratio: 0.20, 95% CI: 0.06–0.67) when other variables including sex, education, and incarceration before 2006 were controlled for (Table 4).

**Table 5:** Crude HIV incidence rates among 2,473 participants followed for 12,140.1 person-years during attendance and non-attendance at MMT.

MMT Status	HIV Incidence	Person-Years	HIV Incidence Rate (Incidence per 1,000 Person-Years)^a^
**In community**	51	6,470.1	7.88 (6.27–9.91)
Attending MMT	3	1,821.2	1.65 (0.66–4.12)
Not attending MMT	48	4,648.9	10.33 (8.16–13.07)
**In prison**	0	5,670.0	0.00

a 95% confidence intervals (in parentheses) were calculated as Wilson score confidence intervals.

### HIV Seroconversions Associated with NSP Utilization

The Poisson regression model indicated that a history of ever obtaining equipment from an NSP (yes or no) was not associated with HIV incidence (incidence rate ratio: 0.58, 95% CI: 0.22–1.54, *p* = 0.2725) after controlling for MMT attendance (Table 6). Although the HIV incidence among participants who reported ever use of equipment from an NSP site for injection was not significantly different from that of participants who never used NSP equipment to inject drugs, frequent users of NSP equipment were at lower risk of HIV infection (no HIV seroconversions occurred among high NSP users). Also, participants who reported obtaining clean needles/syringes from vending machines experienced lower HIV incidence than low NSP users of other NSP sites (Table 7). However, the overall number of participants who seroconverted to HIV was too low to allow statistical significance to be attainable in this subgroup analysis.

**Table 6:** Crude and adjusted association between NSP use and HIV incidence from Poisson regression model with time-dependent variables.

NSP Use	Crude IRR	Adjusted IRR	*p*-Value
Ever NSP	1	1	0.2725
No NSP	0.60 (0.22–1.59)	0.58 (0.12–2.89)	

NSP use treated as a time-varying covariate. The model was adjusted for MMT. 95% confidence intervals given in parentheses.

IRR, incidence rate ratio.

**Table 7:** Crude HIV incidence rates among different NSP use subgroups.

Subgroup	Person-Years	Number HIV of Seroconversions	HIV Incidence (Percent)
Non NSP users	1,726	8	0.5 (0.3–0.9)
High NSP users	337	0	0.0
Vending machine users	306	2	0.7 (0.2–2.0)
Low NSP users	388	6	1.5 (0.8–2.9)

95% confidence intervals (in parentheses) were calculated as Wilson score confidence intervals.

## Discussion

We evaluated the temporal association of the incidence of recently acquired HIV infection among a prisoner population of PWID between 2004 and 2010, and prospectively followed participants in a prison amnesty cohort who were released from prison in Taiwan after the establishment of extensive harm reduction services, to measure the association between use of harm reduction services and HIV incidence among PWID between 2006 and 2010 in Taiwan. Our data indicate that the implementation of a comprehensive harm reduction program—which included MMT, NSPs, and free ART for HIV-infected PWID—was followed by a significant reduction in the HIV incidence rate among PWID in Taiwan. Also, maintaining high coverage of harm reduction services was associated with a sustained low HIV incidence rate and a decreased HIV prevalence among PWID. A few high-income countries have maintained high coverage of several harm reduction strategies for PWID populations. These countries, including Australia and some countries in the European Union, have achieved a stable low HIV incidence rate among PWID that has persisted for years [8],[9]. One study reported that an estimated 50% of opioid injectors in Australia in 2006 and 30% of opioid injectors in the EU in 2004 were covered by OST; additionally, programs issued an average of 385 syringes per PWID per year in Australia and 52 syringes per PWID per year in the EU [9]. In addition, it has been shown in several studies that ART scale-up can lower viral load in the community [26]. Our study population experienced a reversal of an extensive HIV epidemic soon after harm reduction services became available nationwide in Taiwan. Also, an increased proportion of the PWID population who were HIV infected received effective treatment for HIV as the HIV epidemic evolved in Taiwan. It is very likely that reduced HIV infectivity of those receiving ART played a significant role in controlling the epidemic in this population. A successful harm reduction program to reduce HIV incidence and prevalence among PWID after a dramatic increase in HIV incidence has been reported by investigators in Spain [27]. Compared with the response to the HIV epidemic among PWID in Spain, Taiwan implemented comprehensive harm reduction programs very quickly while the HIV outbreak was spreading. These harm reduction programs were associated with effective control of the HIV epidemics in both countries. An active program to detect and treat HIV infections among PWID was important in controlling the spread of HIV among this population in Taiwan.

The data from the amnesty cohort indicate that regular attendance at MMT clinics was associated with a lower HIV incidence among PWID. This finding is supported by previous studies and one meta-analysis study of HIV incidence among PWID, which found a 54% reduction in HIV incidence among PWID who were being treated with methadone replacement therapy [7]. Several studies have reported a lower incidence of HIV among MMT attendees compared to MMT dropouts or non-users [28]–[31]. However, there is evidence of considerable heterogeneity between the different studies included in the meta-analysis [7]. One strength of our study was that we were able to accurately measure MMT use as well as other interventions to prevent HIV transmission in a large population of PWID who were followed prospectively. A comprehensive harm reduction program in Taiwan was scaled up rapidly and was widely used by the PWID population. The prospective follow-up of a cohort of PWID who were tested for HIV at baseline and regularly during follow-up provided a unique opportunity to evaluate the performance of the harm reduction program in preventing the spread of HIV among the population of PWID in Taiwan.

The data from the amnesty cohort also indicate that high use of clean injection equipment was associated with a lower HIV incidence among PWID. Several studies have shown that NSPs reduce HIV infection risk by reducing risky injecting behavior [32]–[35]. However, some systematic reviews of the association of NSPs and HIV incidence have yielded inconsistent results [34]–[36]. This inconsistency is related, in part, to the difficulty in accurately measuring the regularity of use of clean injection equipment from self-report data among PWID. In addition, HIV transmission has been associated with sharing of water, cotton, and other items during the injection of illicit drugs. In addition to the availability of clean injection equipment at NSP sites, unrestricted pharmacy sales of needles or syringes without a prescription are legal in Taiwan. In Taiwan, there are about 6,000 pharmacies. The cost per needle and syringe is only about NT$0.30. Taiwan's NSP also provides alternative access to needles and syringes at pharmacies (pharmacy-based NSP). A systematic review of pharmacy-based NSP programs found insufficient evidence to conclude that these programs provided independent prevention against HIV transmission in the community. Our initial analysis found that NSP use was not associated with a lower HIV incidence among PWID who reported ever using syringes obtained at NSP sites. After this preliminary negative finding from the data obtained after the first follow-up, we modified the questionnaire to obtain more quantitative data on the use of equipment from NSPs by the participants. These data indicated that participants who frequently used equipment from an NSP, i.e., >60% of injection events, had a lower HIV incidence than non-users or occasional users. We also found that participants who reported obtaining syringes from vending machines had a lower HIV incidence than those who never or infrequently used equipment from NSP sites. However, the difference in HIV incidence between these groups was not statistically significant, because the low overall incidence of HIV during the follow-up was not sufficient for subgroup analysis. Since the public health program to control the HIV epidemic among PWID in Taiwan was comprehensive, it is difficult to isolate the independent relationship of a particular component with HIV incidence. Two modeling papers have shown that when ART, NSPs, and OST are brought to scale, each has a strong independent as well as a synergistic effect on lowering HIV incidence [4],[5]. It is possible that PWID engaging in needle sharing or unprotected sex with HIV-positive people who were receiving effective ART may have been protected because the viral load was insufficient to transmit the infection. There was extensive education about the HIV epidemic and the risks of syringe/needle and paraphernalia sharing in the transmission of HIV among PWID in Taiwan in 2005, even before the NSPs were established. Also, prior to the establishment of the extensive network of NSPs, it was easy for PWID to obtain drug injection equipment from pharmacies. However, there were several advantages to PWID of obtaining their equipment from vending machines. First, the machines were convenient and allowed individuals to obtain syringes anonymously at all hours. Also, some participants reported that they had some fear of being identified by the police as a possible heroin user when they attended an NSP site, even after these sites were legalized.

In our study, no incident HIV infections were detected among PWID when they were in prison, although the HIV prevalence among imprisoned PWID was fairly high. Studies in several countries have found drug use and sharing of injection equipment, as well as incident HIV infections, to be common among drug users in prison [37]. A recent study in Scotland found that very infrequent injecting by prisoners when they were incarcerated was associated with a low incidence of HIV infection [38]. Most of the participants in our study cohort reported in interviews that they did not inject drugs in prison. Neither NSPs nor MMT were generally available in prisons in Taiwan prior to the outbreak of HIV. However, the prison system agreed to do a limited evaluation of MMT in a two-prison pilot study after the epidemic was recognized. The interventions to reduce HIV transmission in prisons in Taiwan have included a quarantine strategy (HIV-positive and HIV-negative prisoners live in segregated areas). All new inmates are tested for HIV and each inmate is tested annually. All HIV-positive prisoners are treated with free ART and housed separately from HIV-negative prisoners. In Taiwan, incarceration of PWID is common because heroin use is illegal. Although most PWID reduced or eliminated their use of injecting drugs in prison, they commonly resumed drug use after returning to the community. Several studies have found increases in the likelihood of negative life experiences, such as difficultly finding employment, separation from family, and depression, associated with imprisonment among PWID [39]. Such negative life events, in turn, increase the risk of drug relapse after a prisoner is released from prison.

Our study has some unavoidable limitations. The participants in our study were selected from a group of PWID with histories of incarceration, so they may not be representative of all PWID in Taiwan. Also the data for our analysis relied on the self-selection of participants to utilize or enroll into MMT or NSPs. Thus, as with other observational studies, we cannot be certain that our results are not influenced by other characteristics of the participants that might have affected their risk for HIV infection. Despite this potential limitation, the strengths of our study include the use of data from various interlinked surveillance systems, the use of objective biologic markers, rather than self-reported HIV risk behavior, and successful prospective follow-up of a cohort released into a community with an emerging HIV epidemic among PWID and extensive user-friendly harm reduction services.

In conclusion, the government-initiated extensive harm reduction strategies for HIV prevention in Taiwan have been associated with a substantial reduction in the spread of HIV among PWID and appear to have controlled a rapidly emerging epidemic. Our findings suggest that countries with high prevalence and incidence of HIV among PWID should also offer comprehensive harm reduction services to their populations.

## Abbreviations

ART: antiretroviral therapy
BED assay: BED HIV-1 capture enzyme immunoassay
CDC: Taiwan Centers for Disease Control
MMT: methadone maintenance treatment
NSP: needle and syringe program
OST: opioid substitution therapy
PWID: people who inject drugs
RITA: recent infection testing algorithm
UNAIDS: Joint United Nations Programme on HIV/AIDS
